# A cytoplasmic C-terminal fragment of syndecan-1 is generated by sequential proteolysis and antagonizes syndecan-1 dependent lung tumor cell migration

**DOI:** 10.18632/oncotarget.5174

**Published:** 2015-09-03

**Authors:** Tobias Pasqualon, Jessica Pruessmeyer, Vera Jankowski, Aaron Babendreyer, Esther Groth, Julian Schumacher, Andrea Koenen, Sarah Weidenfeld, Nicole Schwarz, Bernd Denecke, Holger Jahr, Daniela Dreymueller, Joachim Jankowski, Andreas Ludwig

**Affiliations:** ^1^ Institute of Pharmacology and Toxicology, RWTH Aachen University, Aachen, Germany; ^2^ Institute of Molecular Cardiovascular Research, RWTH Aachen University, Aachen, Germany; ^3^ Institute of Molecular and Cellular Anatomy, RWTH Aachen University, Aachen, Germany; ^4^ Interdisciplinary Center for Clinical Research, RWTH Aachen University, Aachen, Germany; ^5^ Department of Orthopaedic Surgery, RWTH Aachen University, Aachen, Germany

**Keywords:** lung cancer, migration, adhesion, proteoglycan, proteolysis

## Abstract

Syndecan-1 is a surface expressed heparan sulphate proteoglycan, which is upregulated by several tumor types and involved in tumor cell migration and metastasis. Syndecan-1 is shed from the cell surface and the remaining transmembrane fragment undergoes intramembrane proteolysis by γ-secretase. We here show that this generates a cytoplasmic C-terminal fragment (cCTF). In epithelial lung tumor A549 cells the endogenously produced cCTF accumulated when its proteasomal degradation was blocked with bortezomib and this accumulation was prevented by γ-secretase inhibition. Overexpression of the cCTF suppressed migration and invasion of A549 cells. This inhibitory effect was only seen when endogenous syndecan-1 was present, but not in syndecan-1 deficient cells. Further, overexpression of syndecan-1 cCTF increased the basal activation of Src kinase, focal adhesion kinase (FAK) and Rho GTPase. This was associated with increased adhesion to fibronectin and collagen G and an increased recruitment of paxillin to focal adhesions. Moreover, lung tumor formation of A549 cells in mice was reduced by overexpression of syndecan-1 cCTF. Finally, delivery of a synthetic peptide corresponding to the syndecan-1 cCTF suppressed A549 cell migration and increased basal phosphorylation of Src and FAK. Our data indicate that the syndecan-1 cCTF antagonizes syndecan-1 dependent tumor cell migration *in vitro* and *in vivo* by dysregulating proadhesive signaling pathways and suggest that the cCTF can be used as an inhibitory peptide.

## INTRODUCTION

Tumor cell migration and invasion critically contributes to tumor spread into distant tissues via blood and lymph [10]. The involved process of cell migration is brought about by the activity of various surface expressed molecules and soluble mediators that stimulate the adhesive behavior and cytoskeletal rearrangement [9, 21, 60]. Syndecans are a family of transmembrane proteoglycans that can critically influence tumor cell migration and invasion [18]. There exist four members of the syndecan family in mammalians (syndecan-1, -2, -3 and -4) [18, 57]. Syndecan-1 is predominantly present on endothelial and epithelial cells and its expression is upregulated in epithelial lung tumors [3, 18, 53]. The core protein of syndecan-1 consists of an ectodomain carrying heparan as well as chondroitin sulfate glycosaminoglycan chains, a single conserved transmembrane domain and a short cytoplasmic domain containing one variable (V) region (specific for each syndecan) flanked by two conserved (C1 and C2) regions [40].

The syndecan-1 ectodomain exerts various protein interactions by direct interaction or binding of extracellular matrix, growth factors, chemokines, cytokines and proteases as reviewed in detail [8, 18, 33, 33]. Besides capturing molecules via glycosaminoglycan chains, the ectodomain of syndecan-1 can cooperate with integrins to maintain cell adhesion and migration [4, 35, 38]. Of note, the cytoplasmic domain of syndecan-1 includes a number of signaling motifs for adapter and signaling molecules involved in cell motility. In fact, the V region of syndecan-1 has been implicated in fascin spike formation and cell spreading [11]. Moreover, the C2 region interacts with PDZ (postsynaptic density, discs large, zona occludens 1 proteins) binding proteins [23, 54]. In addition, a recent report demonstrates that a part of the V and C2 region of syndecan-1 can couple to α_6_ and β_4_ integrin and thereby regulate cell motility [59].

Modulation of focal adhesions by syndecan-1 and integrins was reported during cell migration and focal adhesion formation [5, 26, 35, 42]. Further, activation of focal adhesion kinase and Rho GTPase has been implicated in syndecan-1 mediated effects on cell migration [34, 54]. In addition, syndecan-1 promotes paxillin exchange within the focal adhesion [2]. Moreover, Src kinase phosphorylation and distribution were found to be syndecan-1 dependent [13]. By these pathways syndecan-1 is thought to contribute to tumor cell migration. In fact, tumor cell motility depends on constant assembly and disassembly of focal adhesions [22, 55, 56] and involves the regulation of integrins, focal adhesion kinase (FAK), paxillin, Src and Rho GTPase [20, 39, 42, 55, 58].

Several cell surface expressed molecules implicated in cell migration can undergo proteolytic cleavage at the cell surface (termed shedding) resulting in the loss of the extracellular domain [17, 41]. Also syndecan-1 is shed by several matrix metalloproteinases as well as the disintegrin and metalloproteinase ADAM17 [48]. Clinical studies have demonstrated that the ectodomain of syndecan-1 is released into the blood fluid of lung cancer patients and high levels of release have been associated with poor prognosis and outcome [25, 31, 52]. Several studies have addressed the potential functions of the shed N-terminal ectodomain of syndecan-1. However, much less is known about the remaining C-terminal fragments generated by shedding. We have recently shown that loss of syndecan-1 leads to decreased lung epithelial tumor cell migration *in vitro* and *in vivo* [46]. Reexpression of a syndecan fragment comprising the transmembrane and the cytoplasmic domain (syndecan-1 tCTF) was sufficient to restore migration of these tumor cells suggesting that a promigratory function of syndecan-1 is localized within this fragment. Following ectodomain cleavage, the membrane associated tCTF of syndecan-1 undergoes intramembrane proteolytic cleavage by γ-secretase complex [46].

We here demonstrate that γ-secretase mediated cleavage generates a cytoplasmic syndecan-1 fragment (cCTF) and we ask whether this fragment can still exert specific functions that may be relevant in the context of tumor cell migration. We show that the cCTF can antagonize syndecan-1 mediated cell migration and invasion *in vitro* and *in vivo*. Basal activation of Src, FAK and Rho GTPase is enhanced by the cCTF and this is associated with a more tight adhesion and recruitment of paxillin to focal adhesions suggesting that the cCTF dysregulates adhesive events required for syndecan-1 mediated cell migration. On the basis of these experiments we developed a synthetic syndecan-1 cCTF peptide inhibitor that suppresses syndecan-1 mediated tumor cell migration.

## RESULTS

### Cytoplasmic syndecan-1 cleavage fragments are generated by γ-secretase and degraded via the proteasome

**Figure 1:**
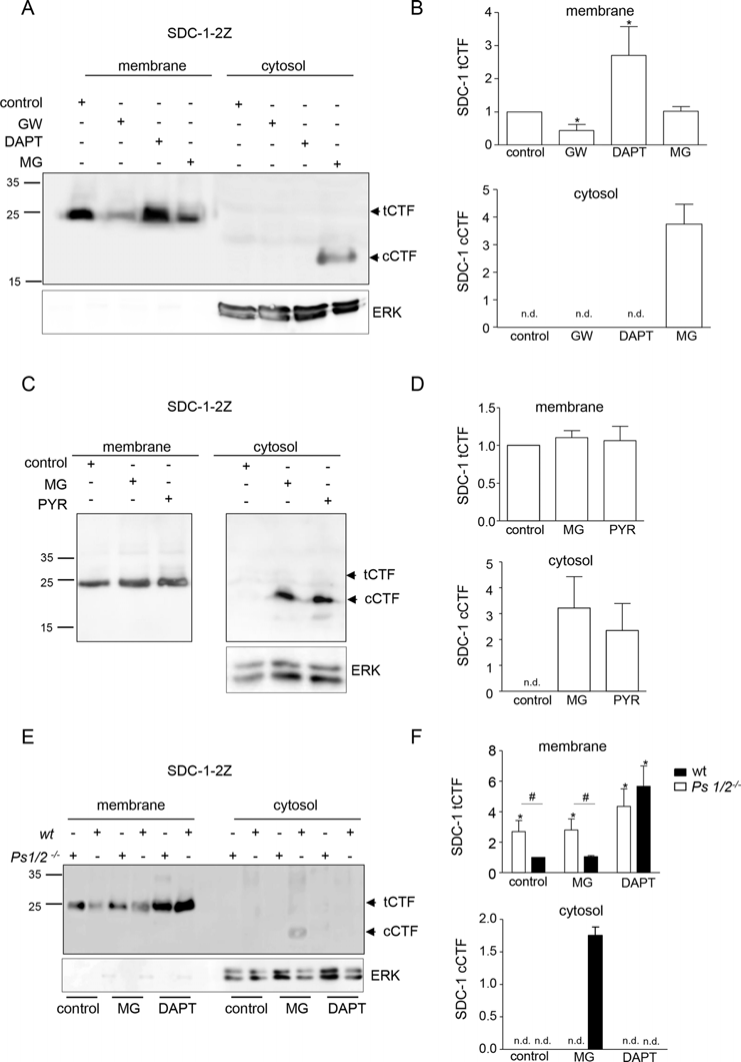
A syndecan-1 cCTFs is generated by γ-secretase and degraded via the proteasome **A, B.** HEK293 cells were transiently transfected with syndecan-1–2Z and treated for 16 h with and without ADAM10/17 inhibitor GW280264X (GW, 10 μM), γ-secretase inhibitor DAPT (5 μM), proteasome inhibitor MG132 (MG, 2 μM) or DMSO (0.01%) as control. The generation of the cell-associated syndecan-1–2Z fragment in the membrane fraction (indicated as tCTF) or the intracellular cytoplasmic syndecan-1–2Z fragment (indicated as cCTF) in the cytosolic fraction was assessed by Western blotting (A) and quantified by densitometry (B). **C, D.** HEK293 cells, transfected with syndecan-1–2Z were treated for 16 h with proteasome inhibitor MG132 (MG, 2 μM), E1 ubiquitin activating protein inhibitor PYR-41 (PYR, 10 μM) or DMSO (0.01%) and investigated by Western blotting (C) The signals were quantified by densitometry and expressed in relation to the signal of the control (D). **E, F.** Wild type (wt) MEF cells and MEF cells lacking the γ-secretase components presenilin 1 and 2 *(Ps1/2*^−/−^) were transfected with syndecan-1–2Z expression vector and subsequently treated with γ-secretase inhibitor DAPT (5 μM), the proteasome inhibitor MG132 (MG, 2 μM) or DMSO (0.01%) for 16 h. The occurrence of syndecan-1–2Z cleavage fragments in the membrane or cytosolic fraction was analyzed by Western blotting (E) and quantified by densitometry (F). In A, C and E, ERK was used as cytosolic marker protein. Data are shown as representative blots and means + SD calculated from three independent experiments. Statistically significant differences compared to the wild type DMSO control, are indicated by asterisk, and differences between wild type and *Ps1/2*^−/−^ are indicated by hashes (*p* < 0.05).

Following ectodomain cleavage by ADAM17 the remaining transmembrane fragment of syndecan-1 undergoes intramembrane proteolytic cleavage by γ-secretase. The latter cleavage should lead to the release of a small fragment into the cytoplasm as it has been described for other γ-secretase substrates including Notch [19]. This was investigated using HEK293 cells transfected with syndecan-1 carrying a C-terminal 2Z-tag. After hypotonic cell lysis, membrane and cytosolic fractions were prepared and analyzed by Western blotting. Normal IgG was used to detect the 2Z-tag via its two binding sites for IgG. While full length syndecan-1–2Z could not be detected due to its high degree of differential glycosylation this technique allowed the detection of tagged syndecan-1 fragments as distinct bands. As expected, syndecan-1 tCTF accumulated in the membrane fraction upon treatment with γ-secretase inhibitor DAPT and disappeared upon treatment with the combined ADAM10 and ADAM17 inhibitor GW280264 (Fig. 1A and 1B), respectively. Interestingly, a small protein fragment with an apparent molecular weight of about 20 kDa became visible in the cytosolic fraction only when cells were treated with the proteasome inhibitor MG132 suggesting that this fragment undergoes proteasomal degradation. This small protein fragment most likely represents a syndecan-1 cytoplasmic C-terminal fragment (cCTF) variant, consisting of the C-terminal 17 kDa tag plus the cytoplasmic C-terminal fragment of syndecan-1.

Accumulation of the syndecan-1 cCTF was also observed upon inhibition of E1 ubiquitin activating ligase using PYR-41 indicating that the cCTF is degraded by an ubiquitin dependent proteasomal pathway. The release of the cCTF into the cytosol was confirmed with cytosolic fractions from wild type murine embryonic fibroblasts (MEFs) transfected with syndecan-1–2Z and treated with and without MG132. The cCTF was not present in the membrane fraction of these cells, which only contained the tCTF (Fig. 1C and 1D). Cytosolic accumulation of syndecan-1 cCTFs by MG132 was not seen in MEFs lacking the crucial γ-secretase components presenilin 1 and 2 (*Ps1/2^−/−^*) (Fig. 1E and 1F). These data suggest that syndecan-1 undergoes sequential cleavage by ADAM17, followed by γ-secretase leading to the generation of a small syndecan-1 cCTF. Despite the finding, that this fragment undergoes proteasomal degradation this does not exclude the possibility that it has functional activity, which may be of pathological and also of pharmacological interest.

### Syndecan-1 cCTF overexpression does not alter gene transcription of lung tumor cells

**Figure 2:**
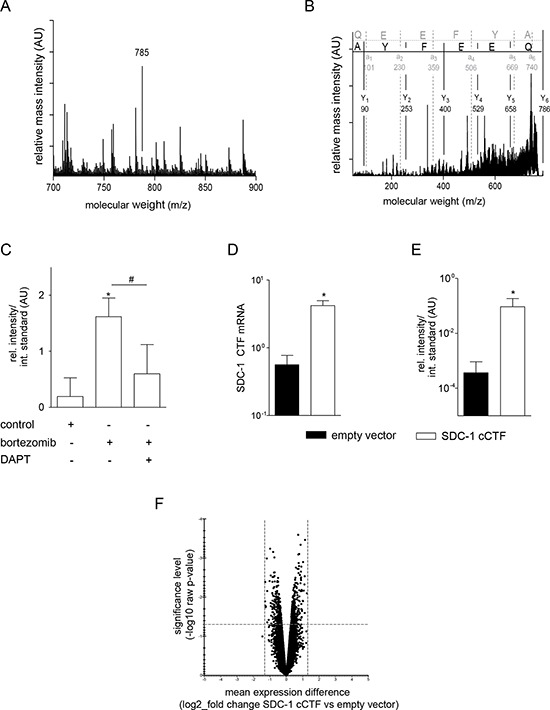
The syndecan-1 cCTF does not affect gene transcription **A, B.** Lysates of A549 cells were subjected to SDS-PAGE. The protein band with the expected size of syndecan-1 cCTF was digested with trypsin and analyzed by MALDI-TOF/TOF. A) Characteristic MALDI-TOF/TOF mass-fingerprint-spectrum: The arrows indicate characteristic mass-signals of peptides corresponding to the tryptic digested syndecan-1 cCTF with a molecular mass of 785 Da. B) Verification of amino acid sequences of the 785 Da peptide by mass spectrometry. **C.** A549 cells were incubated for 16 h in the presence or absence of the proteasome inhibitor bortezomib (5 μM) or γ-secretase inhibitor DAPT (5 μM) and investigated for the presence of endogenous syndecan-1 cCTF by MALDI-TOF/TOF. Quantified mass-signal intensities of the 785 Da peptide were compared to the mass-signal intensities of diinosine pentaphosphate, which was used as internal standard. **D–F.** A549 cells were transduced with lentivirus encoding empty vector or SDC-1 cCTF. D) Efficiency of overexpression was controlled by quantitative PCR with primers annealing to the C-terminus of SDC-1 allowing to detect both endogenous SDC-1 and overexpressed cCTF mRNAs expressed in relation to GAPDH mRNA. E) Mass-signal intensities of syndecan-1 cCTF expressing cells or empty vector control were analyzed by quantitative MALDI-TOF/TOF. F) A549 cells transduced with syndecan-1 cCTF or empty control virus were subjected to microarray gene expression analysis. Results are shown as volcano plot in which each tested gene is indicated as a dot. The *x*-axis represents the log_2_ value of fold change in gene expression between the two groups and the *y*-axis indicates the negative log_10_ raw *p*-value for this difference. Areas top left and top right represent the genes which are up-/down-regulated more or equal to log_2_ 1.3 with a *p*-value lower than 0.05. Data are shown as representative plots and means + SD calculated from three independent experiments. Statistically significant differences compared to the respective control are indicated by asterisks (*p* < 0.05).

To investigate potential functions of syndecan-1 cCTF we used the lung tumor epithelial cell line A549 that predominantly expresses syndecan-1 while other syndecans are expressed at a lower level [1, 7]. To confirm the presence of endogenous syndecan-1 cCTF in these cells, the cell lysate was subjected to SDS-PAGE and the protein band with the expected size of syndecan-1 cCTF was analyzed by tryptic digestion and MALDI-TOF/TOF. The characteristic MALDI-TOF/TOF mass-fingerprint spectrum indicates a mass signal with the expected molecular weight of 785 m/z (Fig. 2A). This signal was due to syndecan-1 cCTF as verified by MALDI-TOF/TOF fragment MS/MS mass-spectra revealing the ultimate N-terminal sequence QEEFYA of the cCTF (Fig. 2B). Moreover, this fragment accumulated after inhibition of the proteasome with bortezomib and this accumulation was prevented by the γ-secretase inhibitor DAPT suggesting that endogenous syndecan-1 undergoes the same processing as described above for the syndecan-1–2Z construct (Fig. 2C). We then used a lentiviral vector to overexpress human syndecan-1 cCTF in wild type A549 cells. Overexpression was controlled by quantitative PCR (Fig. 2D), and the increased presence of protein with the expected molecular mass was confirmed and quantified by mass spectrometry (Fig. 2E).

It is well known for other substrates of γ-secretase such as Notch that the release of their C-terminal intracellular domains into the cytoplasm induces transcriptional responses [32]. Therefore, we controlled whether overexpression of syndecan-1 cCTF would result in the alteration of the overall transcriptional expression profile in A549 cells. However, we could not observe significant gene induction or repression above a 2.5 fold level suggesting that the cCTF by itself does not function to generally control transcription (Fig. 2F). We therefore speculate that the overexpressed cCTF could rather act as a non-transcriptional regulator.

### Syndecan-1 cCTF blocks syndecan-1 dependent lung tumor cell migration and invasion

**Figure 3:**
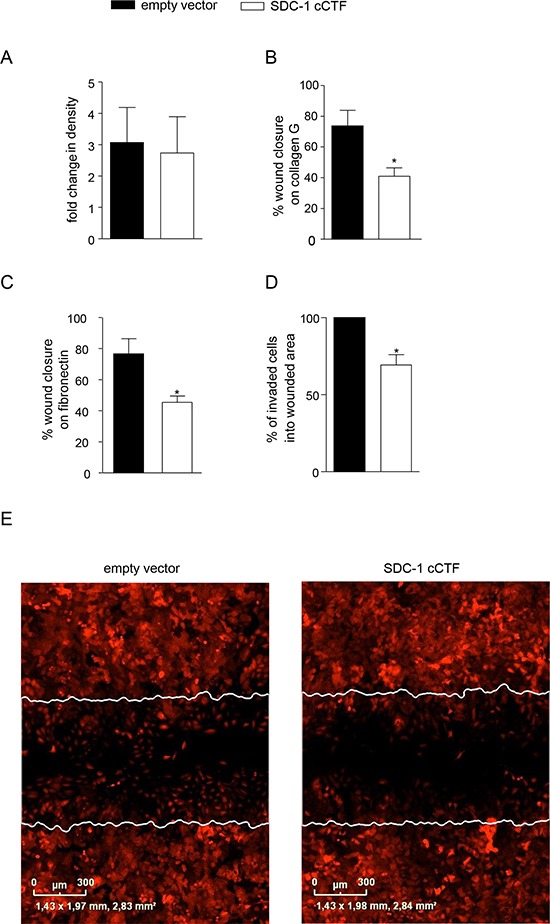
Overexpression of syndecan-1 cCTF blocks cell migration and invasion **A–E.** A549 cells were transduced with lentivirus encoding empty vector or SDC-1 cCTF. Transduced A549 cells were analyzed for proliferation measured as changes in density over 48 h (A) Cells were grown to confluence on collagen G (B) or fibronectin (C) coated wells and wounded by a defined scratch. Wound closure was monitored continuously for 24 h and quantified as percent wound closure in relation to full wound closure using the IncuCyte ZOOM. D–E) Transduced cells were wounded by scratching and subsequently covered with matrigel to analyze cell invasion from the wound edges into the matrigel within the wounded area. Results were expressed as percent of invaded cells in relation to the empty vector control (D). Exemplary images of three independent invasion experiments are shown and the injured area at time point 0 h is indicated by white lines (E). All data were calculated as means + SD from three independent experiments. Statistically significant differences compared to empty vector are indicated by asterisks (*p* < 0.05).

Since syndecan-1 regulates tumor cell migration we questioned whether its proliferative and promigratory function is influenced by the accumulation of syndecan-1 cCTF, which can originate from proteolysis by γ-secretase. Overexpression of the cCTF did not alter proliferation of A549 cells compared to controls (Fig. 3A). By contrast, scratch-induced cell migration on collagen G or fibronectin was significantly reduced upon overexpression of syndecan-1 cCTF (Fig. 3B and 3C). In addition, syndecan-1 cCTF overexpression also reduced invasion of A549 cells into the wounded area covered with matrigel directly after scratch induction (Fig. 3D and 3E). These data indicate that the cytoplasmic CTF of syndecan-1 blocks migration as well as invasion of cells that express endogenous syndecan-1.

**Figure 4:**
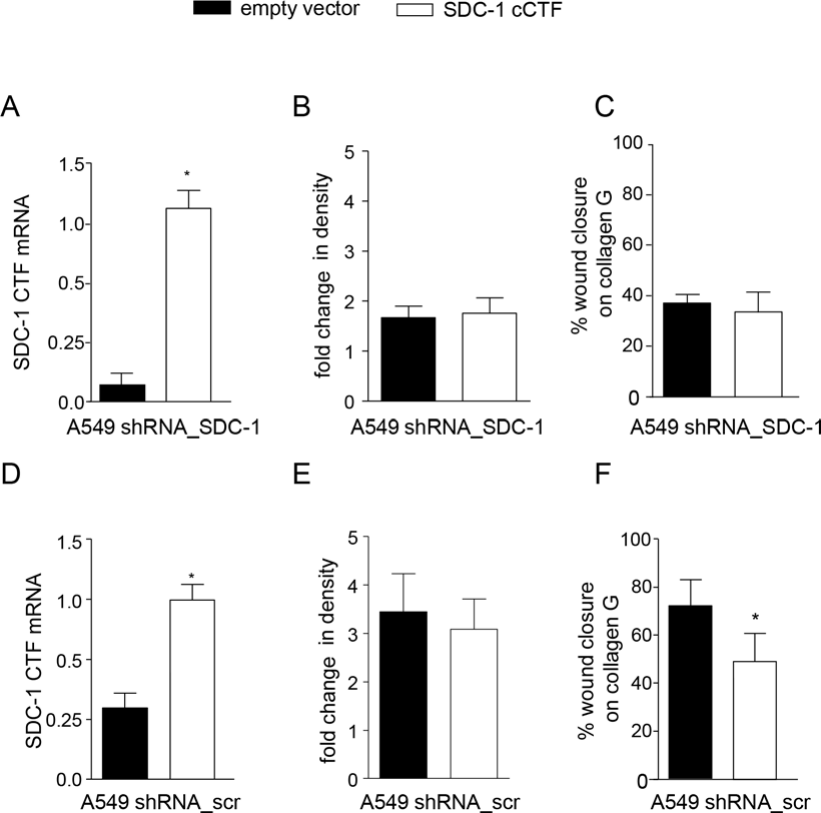
Syndecan-1 cCTF cannot suppress cell migration of syndecan-1 deficient cells **A–F.** A549 cells were transduced with SDC-1 (A–C) or scr shRNA (D–F) Subsequently, a second transduction was carried out with virus for overexpression of SDC-1 cCTF or empty vector control. Efficiency of overexpression was controlled by quantitative PCR with primers annealing to the C-terminus of SDC-1 allowing to detect both endogenous SDC-1 and overexpressed cCTF mRNAs and expressed in relation to GAPDH mRNA (A and D). A549 cells expressing SDC-1 cCTF or empty vector were analyzed for changes in density over 48 h (B and E). Double transduced A549 cells were grown to confluence on collagen G coated wells and wounded by a defined scratch. Wound closure was monitored for 24 h and quantified using the IncuCyte ZOOM (C and F). All data were calculated as means + SD from three independent experiments. Statistically significant differences compared to corresponding empty vector control are indicated by asterisks (*p* < 0.05).

To address the question whether the cCTF can directly antagonize syndecan-1 mediated functions we next investigated whether syndecan-1 cCTF would also influence syndecan-1 independent cell migration and proliferation of A549 cells that were syndecan-1 deficient. First, the importance of syndecan-1 for these responses was confirmed by shRNA mediated silencing of syndecan-1 expression (Supplementary Fig. 1A) leading to significant decrease in cell proliferation and cell migration (Supplementary Fig. 1B and 1C). This finding was consistent with a previous report for A549 cells [46]. Next, wild type syndecan-1 cCTF or empty vector were overexpressed in A549 cells silenced for endogenous syndecan-1 (Fig. 4A–4C) or treated with scramble shRNA (Fig. 4D–4F), respectively. Knockdown of full length syndecan-1 and overexpression of syndecan-1 cCTF was confirmed by quantitative PCR specific for the C-terminal region of syndecan-1 (Fig. 4A and 4D). Syndecan-1 cCTF overexpression showed no effect on proliferation and migration of A549 cells lacking endogenous syndecan-1 (Fig. 4B and 4C). Neither there was an effect on proliferation of cells that were treated with scramble shRNA (Fig. 4E). However, migration of these cells that still produce endogenous syndecan-1 was suppressed when the cCTF was overexpressed (Fig. 4F, compare Fig. 3B). These results suggest that the syndecan-1 cCTF by itself has no promigratory activity but rather counterregulates the promigratory activity of endogenously expressed syndecan-1.

### Syndecan-1 cCTF promotes adhesive events and involved signaling pathways

**Figure 5:**
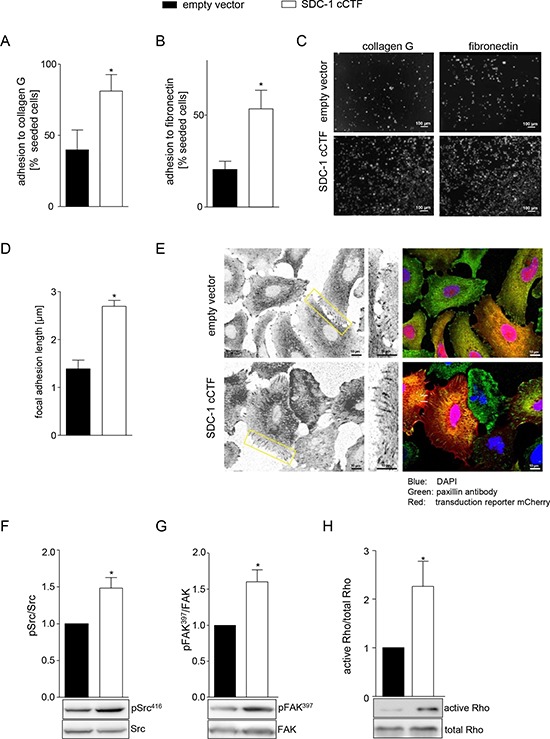
Overexpression of syndecan-1 cCTF promotes adhesive events and related signaling pathways **A–H.** A549 cells were transduced to express SDC-1 cCTF or empty vector. A-C) Transduced cells were investigated for adhesion to collagen G (A) or fibronectin (B). The number of adherent cells was determined using the IncuCyte ZOOM and expressed in relation to the number of seeded cells. Images of a representative cell adhesion experiment to fibronectin and collagen G are shown (C). D-E) Immunocytochemistry of paxillin recruitment. Transduced cells were grown on collagen G, fixed, permeabilized and stained with paxillin antibody (green signal). Expression of mCherry (red signal) was visualized as transduction control. Paxillin recruitment was quantified as lengths of the focal adhesions (D). Focal adhesions are shown as representative images (E). F–H) Cell lysates were analyzed for phosphorylation of Src at Tyr416 (F), FAK at Tyr397 (G) and activation of Rho GTPase (H) by Western blotting. Signals were quantified by densitometry as phosphorylated or activated versus total forms and calculated in relation to the control cells expressing empty vector. Data shown in A, B, D, F–H represent means + SD of three independent experiments and representative Western blots are shown F–H. Statistically significant differences compared to corresponding empty vector control are indicated by asterisks (*p* < 0.05).

The observation that the cCTF influences cell migration led us to investigate whether it would also affect adhesive events. We first studied adhesion to collagen G and fibronectin, which were both increased when syndecan-1 cCTF was overexpressed (Fig. 5A–5C). Since this adhesion is mediated by integrins, we next asked, whether the syndecan-1 cCTF increases or decreases the basal surface expression levels of α_5_, β_1_ (total and active conformation) and β_4_ integrins. However, we observed no changes in their surface expression level (Supplementary Fig. 2A–2H). We then studied focal adhesion length by measuring the recruitment of paxillin (Fig. 5D and 5E). This was significantly increased upon overexpression of the cCTF. We next investigated whether syndecan-1 cCTF overexpression also affects signaling pathways that have been implicated in syndecan-1 dependent adhesion. We previously showed that syndecan-1 regulates phosphorylation of ERK, p38 kinase, Akt and FAK as well as Rho GTPase activation [46]. Overexpression of syndecan-1 cCTF had no effect on phosphorylation of ERK, p38, Akt, and FAK tyrosine 925 (Supplementary Fig. 3A–3D). However, phosphorylation of Src on tyrosine 416 and FAK on tyrosine 397 were significantly increased (Fig. 5F and 5G) compared to the control. Also Rho GTPase activation was increased by syndecan-1 cCTF overexpression (Fig. 5H). These data indicate that syndecan-1 cCTF increases the basal adhesion and also raises the basal activation level of signal transduction pathways, which are involved in focal adhesion formation and cell migration. This disturbance may lead to the suppression of syndecan-1 mediated cell migration.

### Syndecan-1 cCTF suppresses lung metastasis formation in SCID mice

**Figure 6:**
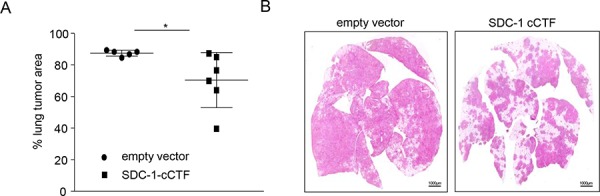
Syndecan-1 cCTF suppresses lung metastasis formation of A549 cells in SCID mice **A–B.** A549 cells were transduced with empty vector or SDC-1 cCTF expression vector. Transduced A549 cells were injected into the tail vein of SCID mice (*n* = 6 per group). After 35 days animals were sacrificed and the lungs were analyzed for lung tumor formation. Lung tumor area was calculated as percentage of total lung tissue area (A) Sections (3 μm) of formalin-fixed and paraffin-embedded whole lungs were stained with hematoxylin-eosin. Representative histologic images are shown (B). All data were calculated as means ± SD and statistically significant differences are indicated by asterisks (*p* < 0.05).

To investigate the *in vivo* relevance of the observed antimigratory activity of syndecan-1 cCTF we used an *in vivo* lung metastasis formation model. A549 cells were injected intravenously into the tail vein of SCID mice and lungs were investigated for tumor metastasis formation. As described previously, tumor formation is suppressed when cells lack endogenous syndecan-1 [46]. We now tested whether inhibition of cell migration by syndecan-1 cCTF would yield a comparable effect. Lung tumor formation of A549 cells transduced to express syndecan-1 cCTF showed significantly reduced lung tumor area compared to cells that had been transduced with empty vector (Fig. 6A and 6B). Hence, the cytoplasmic C-terminal fragment of syndecan-1 can be regarded as an antagonist blocking the functions of endogenous syndecan-1 in lung tumor formation.

### A synthetic syndecan-1 cCTF peptide blocks cell migration and regulates Src and FAK activation

**Figure 7:**
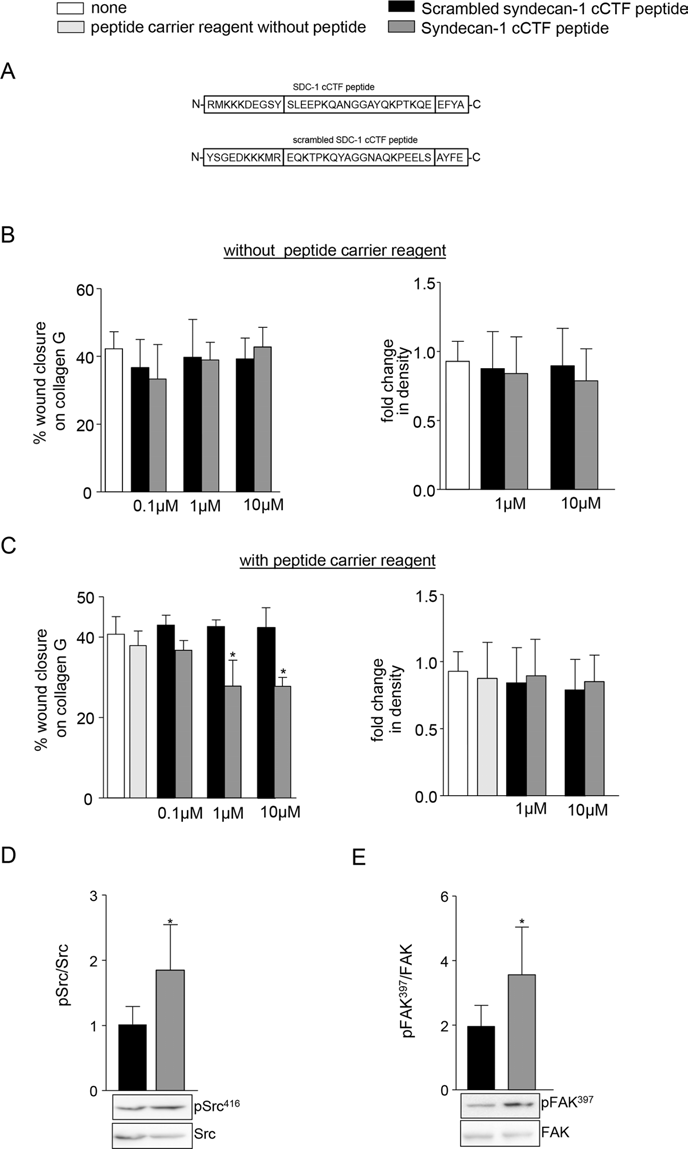
A synthetic syndecan-1 cCTF peptide blocks cell migration **A.** Schematic representation of the human SDC-1 cCTF peptide and the scrambled control peptide. B-D) A549 cells were treated with SDC-1 cCTF peptide or the scrambled peptide without **B.** or with **C.** carrier reagent (Chariot delivery reagent). A549 cells were grown to confluence on collagen G coated wells and wounded by a defined scratch. After wounding, cells were treated with SDC-1 cCTF peptide or scrambled peptide (0.1 μM, 1 μM or 10 μM) and investigated for wound closure over 24 h (left). In parallel, cells were seeded at a lower density and proliferation was quantified as change in density (right) over a period of 24 h using the IncuCyte ZOOM. **D–E.** A549 cells were treated with 10 μM SDC-1 cCTF or 10 μM scramble SDC-1 cCTF with carrier reagent for 2 h. Cell lysates of treated A549 cells were analysed for phosphorylation of Src at Tyr416 (D) and FAK at Tyr397 (E) by Western blotting. Signals were quantified by densitometry as phosphorylated versus total forms and calculated in relation to the control cells. All data were expressed as means + SD and statistically significant differences compared to corresponding scramble syndecan-1 cCTF treatment are indicated by asterisks (*p* < 0.05).

Cell migration is blocked by overexpression of syndecan-1 cCTF in A549 cells. To confirm this finding by an alternative approach we investigated the influence of a synthetic syndecan-1 cCTF peptide on cell migration. We incubated A549 cells with synthetic peptides corresponding to the wild type or a scrambled sequence of syndecan-1 cCTF in the presence or absence of carrier reagent allowing the uptake of the peptides (Fig 7A). Without the carrier reagent both peptides showed no influence on cell migration and proliferation (Fig. 7B) indicating that they do not act as extracellular stimuli or inhibitors for A549 cells. By contrast, in the presence of carrier reagent the syndecan-1 cCTF peptide (1 and 10 μM) significantly decreased cell migration while the scramble control peptide had no effect (Fig. 7C). The proliferative response was not affected by any of the peptides. Furthermore, treated A549 cells showed no changes in morphology compared to controls (Supplementary Fig. 4). We next questioned whether uptake of the syndecan-1 cCTF peptide would also affect migratory signals such as Src and FAK activation. The phosphorylation of Src and phosphorylation of FAK on tyrosine 397 were significantly increased in A549 cells treated with syndecan-1 cCTF peptide compared to the controls receiving the scrambled peptide (Fig. 7D and 7E). The scrambled peptide *per se* did not show an effect compared to the PBS treated control (not shown). This finding confirms that the cCTF counterregulates tumor cell migration and dysregulates signaling pathways involved in cell migration.

## DISCUSSION

Syndecan-1 has been implicated in tumor cell migration and undergoes limited proteolysis at the surface and within the membrane of tumor cells. In the present study we have demonstrated that a cytoplasmic C-terminal syndecan-1 fragment is generated by γ-secretase mediated intermembrane proteolysis. By overexpression of this fragment we show that this cCTF by itself counterregulates syndecan-1 dependent lung epithelial tumor cell migration *in vitro* and *in vivo*. We demonstrate that the overexpressed cCTF interferes with signaling pathways relevant for cell migration and upregulates focal adhesion formation. Moreover, our study shows that a synthetic peptide corresponding to the cytoplasmic C-terminus of syndecan-1 suppresses lung epithelial tumor cell migration and disturbs promigratory signaling. Thus, we here provide pharmacological and genetic evidence that the cCTF can antagonize promigratory effects of syndecan-1. Our study indicates that the antimigratory activity of the cCTF may be useful to limit prometastatic effects of syndecan-1.

Syndecan-1 has been found to promote migration of epithelial lung tumor cells. But also a suppressive function in migration of breast cancer epithelial cells and keratinocytes has been described [26, 46, 59, 63]. Moreover, there exist several mechanisms by which syndecan-1 can potentially influence tumor growth and spread. The ectodomain can bind and promote the activity of growth factors, chemokines and proteases and thereby contribute to cell growth and migration [8, 18, 33, 33]. Further, intracellular protein interaction may represent a mechanism influencing cell migration. In a previous study, we have shown that silencing of endogenous syndecan-1 reduces migration and invasion of cultured A549 cells. This was correlated with a reduced ability of these cells to form lung tumors in SCID mice [46]. Importantly, both *in vitro* and *in vivo* activity could be reconstituted by a syndecan-1 variant lacking the ectodomain. These data suggest that a promigratory activity is localized within the C-terminal part of syndecan-1. However, in the present study a smaller fragment only consisting of the cytoplasmic portion of syndecan-1 turned out as an inhibitor of cell migration. In fact, inhibitory effects on cell migration by partial structures of intracellular syndecan-1 domains have been reported recently [59]. A cell-penetrating peptide containing the C2 domain and parts of the variable region of syndecan-1 was found to disrupt HER2-dependent epithelial cell haptotaxis. We here describe a different antimigratory peptide consisting of the full intracellular domain of syndecan-1. We show that this peptide is generated by limited proteolysis within the cell membrane and therefore may play a role as a natural inhibitor of cell migration.

Several surface proteins with a single transmembrane domain undergo intramembrane proteolysis upon their proteolytic shedding at the cell surface. The analysis of potential functions of cytoplasmic cleavage fragments arising from intramembrane proteolysis is complicated by the fact that these fragments can undergo further proteolysis via the proteasome as it has been described for the chemokines CX3CL1 and CXCL16 [50], the cytokine receptor IL6R [12] or the amyloid precursor protein APP [43]. In fact, also the syndecan-1 cCTF can undergo ubiquitin dependent degradation via the proteasome. However, this does not exclude the possibility that the cCTF by itself can fulfil a function within the cell. Such potential function may become more relevant under conditions of reduced proteasome activity which occurs upon treatment with drugs blocking the proteasome such as bortezomib [15] or genetic proteasomal disorders [30]. A proteasome-regulated function of the intracellular cleavage fragment is well known for Notch [44]. Notch is sequentially cleaved by ADAMs and the γ-secretase complex [19]. This leads to the intracellular release of a C-terminal fragment into the cytoplasm, which is a critical step for its nuclear transport and transcriptional activity during development and hematopoiesis. Intramembrane proteolysis by γ-secretase was also found to contribute to the transcriptional regulation induced by CD44 and the MHC class II chaperone CD74 [6, 47]. Compared to the cCTF of Notch, CD44 and MHCII, the C-terminus of syndecan-1 cCTF is relatively small and it does not contain motifs that suggest a transcriptional activity. Nevertheless, following its overexpression syndecan-1 was found to translocate into the nucleus in human fibrosarcoma cells and this was dependent on a portion of the cytoplasmic C1 domain with the amino acid sequence RMKKK [63]. By this mechanism syndecan-1 was proposed to upregulate proadhesive and promigratory genes. However, in our setup we could not obtain evidence that such a possible mechanism is used by the syndecan-1 cCTF. In fact, our transcriptional analysis did not indicate significant changes on transcriptional level upon syndecan-1 cCTF overexpression. Therefore, the suppressive effect of the syndecan-1 cCTF on cell migration seems to involve posttranslational rather than transcriptional mechanisms. Of note, the proteasomal inhibitor bortezomib, which is used in the clinic to prevent transcription factor activation (e.g. NF- κB) in multiple myeloma cells, causes the accumulation of the syndecan-1 cCTF in tumor cells [14]. Such a process would reduce tumor cell migration and could be beneficial for the treatment.

The cytoplasmic proportion of syndecan-1 can interact with various adapter proteins. The highly conserved C2 region of syndecan-1 contains a PDZ binding motif and interacts with PDZ binding proteins [23, 54]. Moreover, the cytoplasmic domain of syndecan-1 was found to interact with the intracellular domain of β_4_-integrin and thereby regulate cell adhesion. This was mediated by the C2 domain plus part of the adjacent variable region of syndecan-1. In our study, overexpression of the syndecan-1 cCTF had no direct effect on integrin surface expression. Yet, adhesion and related processes such as paxillin recruitment to focal adhesions increased when the cCTF was overexpressed. In fact, syndecan-1 is well known to contribute to focal adhesion formation [5, 26, 35, 42] and has been reported to promote paxillin exchange within the focal adhesion [2]. Moreover, basal activation of signaling proteins implicated in cell adhesion such as FAK phosphorylation at Tyr397 as well as Rho GTPase activation were found to be enhanced by the cCTF. And indeed, activation of FAK and Rho GTPase have been implicated in syndecan-1 mediated effects on cell migration [34, 54]. Of note, FAK phosphorylation at site Tyr925, which would normally drive exclusion of the kinase from focal adhesions was not affected by the cCTF [24, 37]. Finally, the cCTF also enhanced basal Src phosphorylation at Tyr 416 which has been reported to be syndecan-1 dependent [13]. All these syndecan-1 mediated responses are known to drive tumor cell migration [20, 39, 42, 55, 58]. Thus, in our experimental setup the syndecan-1 cCTF critically influences proadhesive signaling events, which are syndecan-1 dependent and required for tumor cell migration. The observed upregulation of constitutive signaling by FAK and Src may directly cause more mature focal adhesion contacts and may finally lead to stronger adhesion. As the formation and resolution of focal adhesions is a critical step in cell migration this dysregulation of cell adhesion by the cCTF may then result in the defect in cell migration. Such a dysregulation could arise from an intracellular interaction of the cCTF with signaling proteins that normally bind to intact syndecan-1 such as PDZ binding proteins [23, 54] or integrins [59]. Since we found that syndecan-1 cCTF only exerts its inhibitory function on cell migration when endogenous syndecan-1 is present, it may be possible that the cCTF competes with the full length syndecan-1 for intracellular interaction partners and thereby reduces signaling of syndecan-1. In addition, there may be interference between the various types of syndecans, which can form hetero-oligomers [62]. For example syndecan-2 was demonstrated to interact with syndecan-4 via the transmembrane domain. Interestingly, when both syndecans were coexpressed in colon cancer cells, the syndecan-2 mediated promigratory and adhesive effect was decreased by syndecan-4 and *vice versa*. It remains to be determined whether also syndecan-1 can influence other syndecans by forming hetero-oligomers and whether such interaction is regulated by the proteolytic processing of the syndecans [16].

High serum levels of syndecan-1 were observed in patients with lung epithelial tumors of poor prognosis suggesting that syndecan-1 may represent a versatile tumor marker [25, 31, 52]. Besides this, syndecan-1 may also be regarded as a target for the treatment of some tumor types. This is indicated by several reports showing that syndecan-1 contributes to tumor growth *in vivo*. Syndecan-1 deficient mice were found to develop less lung tumors when treated with carcinogens [36]. Syndecan-1 has also been proposed as target in myeloma therapy [61]. As a pharmacological approach an inhibitory antibody against syndecan-1 has been developed and successfully used to block melanoma growth and ovarian carcinoma [45]. Inhibitory peptides may represent an alternative approach as shown for synstatin, which competitively displaces the integrin and IGF1R kinase from the syndecan-1 and thereby inhibits tumorgenesis and angiogenesis [49]. By this it may be possible to target selected functions of syndecan-1 mediated by its different domains. These peptide inhibitors against the cytoplasmic portion of syndecan-1 may serve to selectively target the intracellular promigratory activity of syndecan-1, without affecting its extracellular function. In the future, side effects of these approaches on wound healing and inflammatory responses need to be studied in detail.

## MATERIALS AND METHODS

### Recombinant proteins, antibodies, fluorescent dyes, and inhibitors

Rabbit polyclonal antibody (Ab) against human ERK (C-16), mouse monoclonal Ab (mAb) against human phospho- (p-) ERK (Thr202/Tyr204) (clone E-4), rabbit Ab against human FAK, rabbit Ab against human p-FAK (Tyr925) and rabbit Ab against human c-Src (sc-18) were from Santa Cruz Biotechnology (Dallas, TX, USA). Rabbit mAb against human Akt (clone C67E7), rabbit mAb against human p-Akt (Ser473) (clone D9E), polyclonal rabbit Ab against human p38, mouse mAb against human p-p38 (Thr180/Tyr182) (clone D3F9) and rabbit Ab against human p-Src (Tyr416) (clone 2101) were from Cell Signaling (Danvers, MA, USA). Rabbit mAb against human p-FAK (Tyr397) (clone 141–9) was from Invitrogen (Karlsruhe, Germany). Mouse mAb against human paxillin (clone 349/paxillin) was from BD Biosciences (Heidelberg, Germany). Allophycocyanin- or peroxidase-conjugated secondary antibodies were from Jackson (Newmarket, UK) and goat Ab conjugated with AlexaFluor^®^488 against mouse IgG was from Life Technologies (Carlsbad, CA, USA). Antibodies were used according to the manufacturers' recommendation. The metalloproteinase inhibitor GW280264 was synthesized and assayed for inhibition of recombinant human ADAM17 and ADAM10 as described [27]. The proteasome inhibitor MG132, the γ-secretase inhibitor DAPT (*N*-[*N*-(3,5-difluorophenacetyl-L-alanyl)]-*S*-phenylglycine *t*-butyl ester) and the ubiqutin ligase E3 inhibitor PYR-41 were from Calbiochem (Darmstadt, Germany). The proteasome inhibitor Velcade^®^ (bortezomib) was from Millennium Pharmaceuticals Inc. (Cambridge, USA).

### Cell culture and transfection

All cells were cultured in DMEM supplemented with 10% fetal calf serum and 1% penicillin/streptomycin (all from Sigma-Aldrich). The lung tumor epithelial cell line A549 and the human embryonic kidney cell line HEK293T were cultured as described [48]. Presenilin 1/2 double-deficient (*Ps 1/2*^−/−^) and wild type murine embryonic fibroblasts (MEF) from murine embryos were provided by Paul Saftig and Karina Reiss (University of Kiel, Germany) and cultured as described [51]. Transient transfection was carried out with Lipofectamine 2000 (Invitrogen) according to the manufacturer's instructions.

### DNA constructs

Short hairpin RNA (shRNA) targeting human syndecan-1 was inserted into the lentiviral expression vector pLVTHM-GFP using MluI and ClaI [48]. The targeting sequence was *ggacttcacctttgaaacc* (syndecan-1–1016; target: CDS, extracellular domain of syndecan-1 mRNA) as described previously [46]. A sequence of *ccgtcacatcaattgccgt* served as scramble control. The human syndecan-1 with 2Z-binding domain was constructed as described previously [28, 46]. For overexpression of the cCTF, synthetic human cDNAs encoding the Kozak sequence directly followed by the cytoplasmic C-terminal fragment (consisting of the C1, V and C2 domain) of syndecan-1 (amino acid position 276–310) was synthesized by MWG Biotech and inserted into the lentiviral expression vector pLVX-IRES-mCherry (Clontech, Mountain View, CA, USA) using EcoRI and NotI.

### Lentiviral transduction

The lentiviral production and transduction of target cells was carried out as described previously [48]. For shRNA mediated silencing of syndecan-1, the lentiviral vector system pLVTHM was used. For overexpression of syndecan-1 cCTF in wild type A549 cells the pLVX system from Clontech was used. For overexpression of cCTF or empty vector in scramble or syndecan-1 deficient cells a second transduction with lentiviral particles was carried out 5 days post-transduction with lentiviral shRNA particles. The transduction efficiency was analyzed by GFP (shRNA expression) or mCherry (overexpression) reporter gene expression 72 h after transduction using fluorescence microscopy.

### Quantitative PCR analysis

Syndecan-1 CTF expression was quantified by quantitative PCR and normalized to the mRNA expression level of glyceraldehyde-3-phosphate dehydrogenase (GAPDH) as described previously [46]. The following primers were used: syndecan-1 CTF forward, *aggacgaaggcagctac*; syndecan-1 CTF reverse, *gcctggtggggtttctggtag*; GAPDH forward, *ccagtgagcttcccgttca*; GAPDH reverse, *cagaacatcatccctgcctcta*. All PCR reactions were run on a LightCycler 480 System (Roche) with 45 cycles of 10 s denaturation at 95°C, followed by 10 s annealing at 54°C (syndecan-1 CTF) or 66°C (GAPDH) and 15 s amplification at 74°C. Standard curves for target genes and reference gene (GAPDH) were prepared from a serial dilution of pooled cDNA products of all samples. Data were obtained as the crossing point value normalized according to the e-method using the LightCycler^®^480 software 1.5.

### Western blot analysis of 2Z-tagged syndecan-1

The Western blot analysis of 2Z-tagged syndecan-1 was performed as described previously [46]. 2Z-tagged syndecan-1 was detected via the IgG binding sites of the 2Z-tag by probing with normal rabbit IgG (0.5 μg/ml).

### Cytosolic and membrane fractions

To separate the membrane and cytosolic fraction, 5 × 10^6^ transfected HEK293 cells were suspended in 1 ml hypotonic 5 mM HEPES buffer, pH 7.4, passed through a 27G cannula for 15 times and centrifuged at 700 × g for 20 min. Subsequently, 100 μl 1.4 M NaCl was added to 900 μl supernatant and membranes were sedimented at 20,000 × g for 60 min. Pellets were solved in 2-fold concentrated reducing Laemmli buffer, supernatants were concentrated 5-fold using 3 kDa cut-off Vivaspin 500 ultrafiltration devices (Sartorius, Goettingen, Germany), and 5-fold concentrated Laemmli buffer was added.

### Phosphorylation analysis of ERK, p38, Akt, Src and FAK

A549 cells (3.0 × 10^6^ cells per well, 6-well plate) were cultured for 24 h, cooled on ice and lysed in lysis buffer containing 20 mM Tris, 150 mM NaCl, 5 mM EDTA, 30 mM NaF, 5 mM DTT, 1 mM PMSF, 10 mM pNPP, 1 mM benzamidine, 10 mM glycerophosphate, 1 mM Na_3_PO_4_, 1% Triton X-100, and Complete protease inhibitor (Roche). Lysates were then subjected to SDS-PAGE and Western blotting using antibodies against phosphorylated and non-phosphorylated forms of ERK, p38, Akt, Src and FAK. Antibodies were used according to the manufacturer's recommendations.

### Rho GTPase activation assay

A549 cells (3.0 × 10^6^ cells per well) were cultured for 24 h and subsequently cell lysates (500 μg total protein) were analyzed for content of total and active Rho GTPase using a commercial kit (Enzo Life Sciences, Plymouth, USA) according to the manufacturer's recommendations.

### Adhesion assay

The adhesion assay was carried out in 48-well plates. Wells were coated with 125 μl fibronectin (12 μg/ml) or collagen G (40 μg/ml) for 1 h at room temperature or 37°C. After the incubation, the coated wells and uncoated controls were blocked with 200 μl 0.5% BSA (diluted in HBSS) for 15 min at room temperature and subsequently washed twice with HBSS. Transduced A549 cells were resuspended in HBSS and 100 μl (1 × 10^5^ cells) cell suspensions were added to the coated wells, sedimented for 30 s at 300 × g and incubated for 15 min at 37°C, respectively. Subsequently, the wells were washed several times and the fluorescence signals of adherent cells were analyzed using the *IncuCyte ZOOM microscope* (Essen Biosciences, Hertfordshire, UK).

### Proliferation assay

For live-cell analysis of proliferation, 5 × 10^3^ cells in 100 μl/well were seeded in 96-well plates. Cell proliferation was monitored using the automated *IncuCyte ZOOM microscope* by taking images of each well every 2 h for a period of 48 h as described previously [46]. The calculation of density was performed with the *IncuCyte ZOOM microscope* software 2014A. Results were expressed as fold increase in density after 48 h in comparison to 0 h.

### Wound closure assay (scratch assay)

For live-cell analysis of scratch-induced cell migration, 1.5 × 10^4^ cells per well were seeded on collagen G (40 μg/ml) or fibronectin (12 μg/ml) (Biochrom AG, Germany) coated 96-well plates near confluence and allowed to grow overnight in standard medium. At confluence, cells were pretreated 2 h with mitomycin (10 μg/ml) (Medac, Germany) to block cell proliferation and washed with standard media. Subsequently, a defined scratch (wound width between 642–767 μm) was performed in each well using the certified *Essen Bioscience automated 96-wound-maker™* (Essen Bioscience, Hertfordshire, UK) for 96 well-plates. The medium was removed and 100 μl standard medium was added to the wells. The closure of the wounded area was monitored using the *IncuCyte ZOOM* system by taking images of each well every 2 h over a period of 24 h. The reduction of wound width was determined over time using the *IncuCyte ZOOM microscope* software 2014A. Data were expressed as percentage of wound closure after 24 h.

### Invasion assay

After scratch induction (see above), the medium was removed and 40 μl matrix basement membrane matrigel (BD Biosciences) was added to the wells. The invasion of cells into the wound field was recorded using the *IncuCyte ZOOM system*. The time range of measurement was from 0 h to 48 h after scratch induction. The number of invaded cells in the wound area was determined and calculated as percentage of cells present before scratch induction.

### Immunocytochemistry

For paxillin immunofluorescence staining, syndecan-1 cCTF or empty vector expressing cells were grown onto glass coverslips and fixed with 4% paraformaldehyde for 20 min at room temperature. The fixed cells were probed with the paxillin antibody followed by AlexaFluor^®^488 FITC secondary antibody. Images were taken with an ApoTome.2 (Zeiss, Goettingen, Germany) with a plan-apochromat 63x/1.4 Oil objective (Zeiss) using AxioVision LE software (Zeiss). Length of focal adhesions was quantified using the measure option in ImageJ 1.49s software. Three independent experiments were performed of each condition and at least 70 focal adhesions were measured.

### Trypsin digestion of syndecan cCTFs and Matrix-Assisted Laser Desorption/Ionization-time of flight- (MALDI TOF/TOF)

Cell lysates of transduced A549 cells were subjected to 15% SDS-PAGE under reducing conditions. Proteins were stained with Coomassie Brilliant Blue G 250. Polypeptide bands of interest were cut out manually and plugs were washed/equilibrated with ammonium bicarbonate in acetonitrile. Afterwards, the plugs were digested with 0.05 μg trypsin. The resulting peptides were desalted utilizing the ZipTip™ (Millipore, Billerica, MA, USA) technology and eluted with 80% acetonitrile directly onto the MALDI target plate using a-cyano-4-hydroxycinnamic acid as matrix. The subsequent mass spectrometric (MS) analyses were performed with a MALDI-time of flight/time of flight (TOF/TOF) mass spectrometer (Ultraflex III; Bruker-Daltonic, Germany). Calibrated and annotated spectra were subjected to a database search (Swiss-Prot, Zürich, Switzerland) utilizing Bruker's Bio- Tools 3.2 and the Mascot 2.3 search engine (Matrix Science Inc, Boston (MA), US) to compare the experimental MS as well as MS/MS data set and the calculated peptide mass-signal for each entry in the sequence database and empirically determined factors to assign a statistical weight to each individual peptide match. Quantification of the mass-signal intensities of syndecan-1 cCTFs were compared to the mass-signals intensities of diinosine pentaphosphat used as internal standard.

### Production of synthetic syndecan cCTF peptides

Syndecan cCTF peptides were synthesized by ThermoScientific (Ulm, Germany). Syndecan-1 cCTF amino acid sequence was (YR MKK KDE GSY SLE EPK QAN GGA YQK PTK QEE FYA). A scramble syndecan-1 cCTF with the amino acid sequence (ED KKK MRY E QKT PKQ YAG GNA QKP EEL SYS G AYFE) was used as control. Lyophilized synthetic peptides were reconstituted in sterile water at a concentration of 0.1, 1.0 and 10 μM.

### Synthetic syndecan cCTF peptide carrier reagent

Peptide delivery was carried out using the *Chariot™* peptide carrier reagent (Active Motif, Rixensart, Belgium) according to the manufacturer's recommendations. Briefly, for peptide delivery in cells, synthetic peptides were diluted in PBS and mixed with an equal volume of 1/25 diluted Chariot transfection reagent in sterile water. The mixture was incubated at room temperature for 30 min to allow complex formation. Cells grown in 96-well plates were overlaid by 20 μl mixture and 80 μl of serum-free medium was added. Cells grown in 6 well plates were overlaid with 200 μl mixture and 1.5 ml serum-free medium was added.

### GeneChip^®^ hybridization

Gene expression in cells expressing shRNA against SDC-1 or shRNA scramble as control were analyzed in independent experiments using the GeneChip^®^ Human Transcriptome Arrays 2.0 (Affymetrix, Santa Clara, CA, USA). Total RNA was isolated using RNeasy Kit (Qiagen) and quantified. Subsequently, RNA quality was assessed using RNA 6000 Nano Assay with the 2100 Bioanalyzer (Agilent, Santa Clara, CA, USA) to ensure that the samples had a RNA integrity number (RIN) of at least 9. Samples, each 250 ng total RNA, were prepared and hybridized to the GeneChip^®^ Human Transcriptome Arrays 2.0 according to the GeneChip^®^ WT Plus Reagent Kit user manual (P/N 703174 Rev. 2; Affymetrix). Processed samples were hybridized to GeneChip^®^ Human Transcriptome Arrays 2.0 at 45°C for 16 h with 60 rpms, washed and stained on a Fluidics Station 450 (program: FS450 0001) and scanned on a GeneChip^®^ Scanner 3000 7G (both Affymetrix). To assess differential expression, CEL files were normalized by robust multiarray average (RMA [29]) to facilitate comparisons across arrays using the AltAnalyze 2.0.9 software suite (Gladstone Institutes, San Francisco, CA, USA). Default analysis options (analysis of gene expression *via* a rawp) were used.

### Lung tumor metastasis

The lung tumor metastasis model was carried out as described previously [46]. A549 cells were studied for metastasis formation in severe combined immune deficient mice (NOD-SCID *Il2rg*^null^, NSG) that were maintained under specific-pathogen-free conditions in the central animal facility of the University Hospital RWTH Aachen. All animal experiments were approved by local authorities in compliance with the German animal protection law (AZ 84–02.04.2013.A198). A549 cells were harvested, singularized, washed and resuspended in PBS. Subsequently, a uniform single cell suspension containing 3 × 10^6^ cells in 100 μl of PBS was intravenously injected into the lateral tail vein of 6–8 week-old SCID mice. After 35 days, lungs were prepared, fixed by intratracheal instillation of Roti-Fix^®^ (Roth, Germany), embedded in paraffin and cut in 3 μm slices. Hematoxylin-eosin staining was performed using standard protocols. Images were taken with a Zeiss microscope (AxioLab.A, Carl Zeiss MicroImaging GmbH, Germany) at 40-fold magnification. For mounting whole lung overviews the Keyence BZ-9000 software BZ2Viewer Merge was used. Tumor area was measured using ImageJ software 1.48v (Rasband, NIH, Bethesda, Maryland, US) and denoted as percentage of whole lung tissue area.

### Statistics

Percentage data were arc-sin transformed. Statistics were calculated using PRISM5.0 (GraphPad Software, La Jollla, CA). Where applicable, data were analyzed by Student's *t*-test and differences were indicated by asterisk or hashes. To test for difference to a single hypothetical value (e.g. 100 or 1.0) the one-sample *t*-test was used and differences were indicated by asterisks or hashes. *P*-values were corrected for multiple comparisons using false discovery rate (FDR).

## SUPPLEMENTARY FIGURES


