# Supplementary material for: A cytoplasmic C-terminal fragment of syndecan-1 is generated by sequential proteolysis and antagonizes syndecan-1 dependent lung tumor cell migration
Source: Oncotarget. 2015 Sep 3;6(31):31295–312. doi: 10.18632/oncotarget.5174 (PMC4741606; doi:10.18632/oncotarget.5174)
Supplement: Supplementary file 1 [file oncotarget-06-31295-s001.pdf]

SUPPLEMENTARY FIGURES

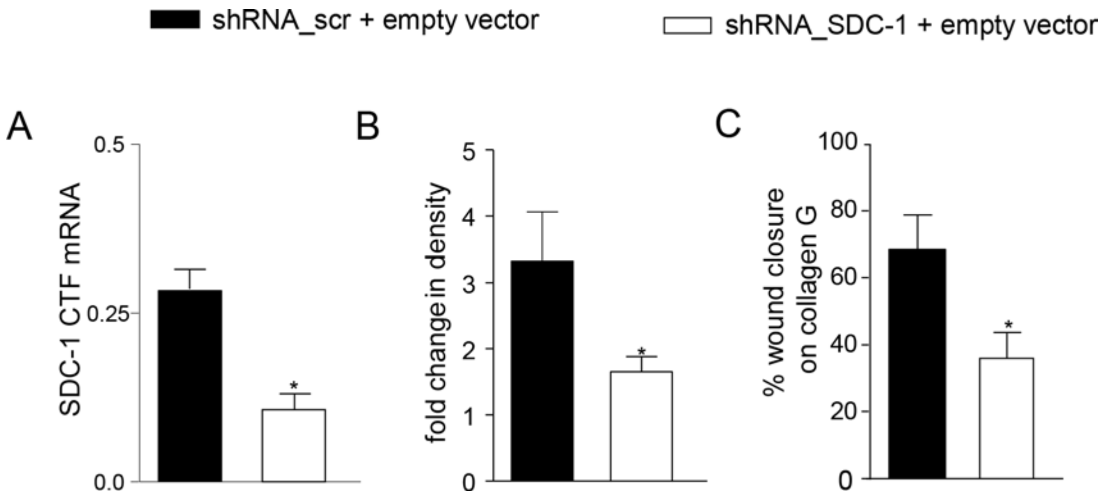

Supplementary Figure S1: Loss of syndecan-1 reduces cell migration in A549 cells. A–C. A549 cells were transduced with lentivirus encoding scr or SDC-1 shRNA. SDC-1 mRNA knockdown was controlled by quantitative PCR (A). Transduced cells were analyzed for proliferation measured as fold change in density over 48 h (B). Cells were grown to confluency on collagen G coated wells and wounded by a defined scratch. Wound closure was monitored for 24 h and quantified using the IncuCyte ZOOM (C). All data were calculated as means + SD from three independent experiments. Statistically significant differences compared to scr transduced cells are indicated by asterisks ($p < 0.05$).

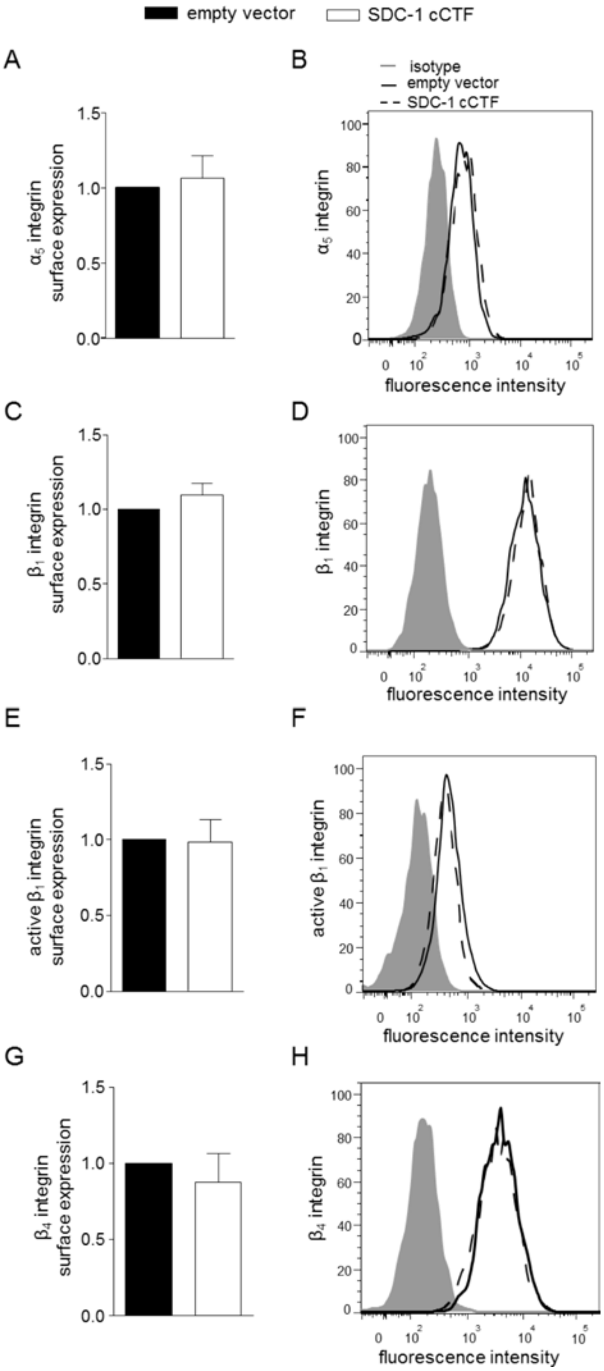

Supplementary Figure S2: SDC-1 cCTF expression does not modulate integrin surface expression. A–H. A549 cells were transduced with empty vector or SDC-1 cCTF expression vector. Transduced A549 cells were investigated for α_5 integrin (A and B), β_1 integrin (C and D), active β_1 integrin (E and F) and β_4 integrin surface expression (G and H) by flow cytometry. Data were quantified as relative changes of the fluorescence signal compared to the empty vector control and represent means + SD of three independent experiments (A, C, E and G). Representative histograms are shown (B, D, F, H). No statistically significant differences were detected.

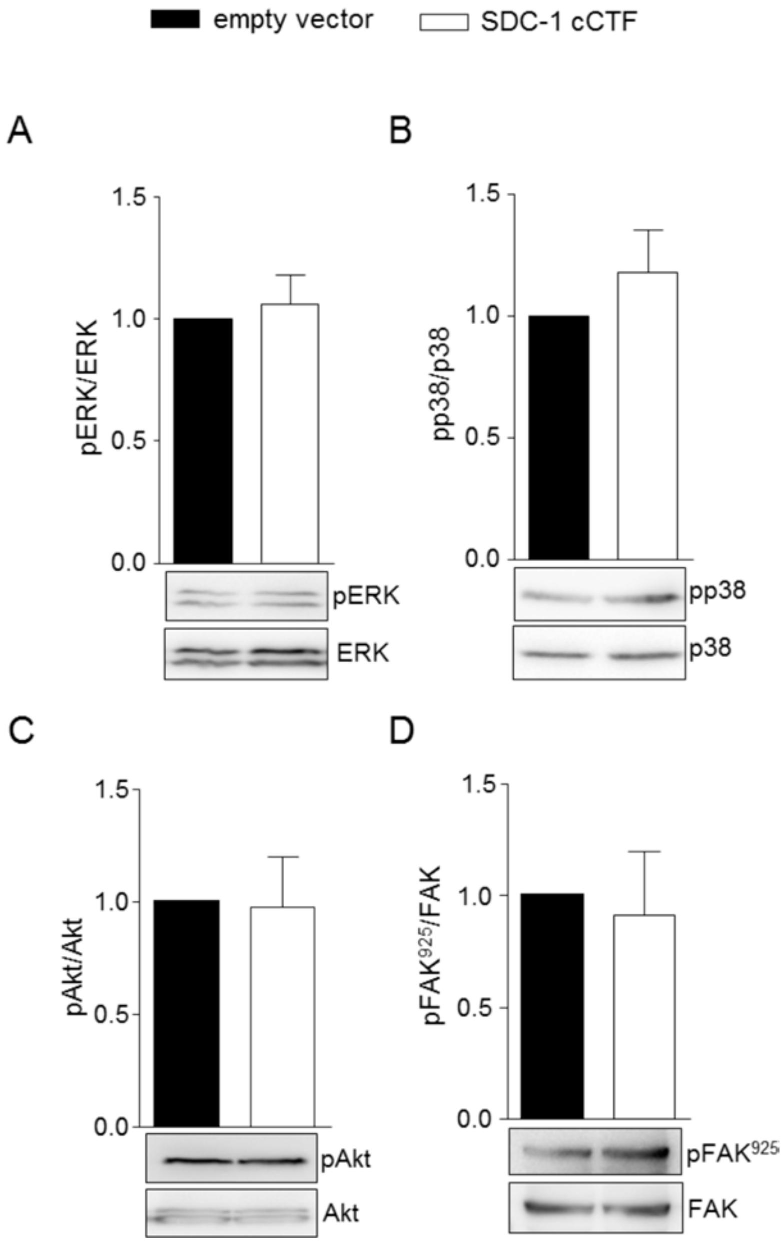

Supplementary Figure S3: SDC-1 cCTF expression does not regulate ERK, p38, Akt or FAK^{Tyr925}. A–D. Cell lysates of A549 cells transduced to with empty or SDC-1 cCTF expression vector were analysed for phosphorylation or basal expression status of ERK (A), p38 (B), Akt (C), FAK site Tyr925 (D) by Western blotting. Signals were quantified by densitometry as phosphorylated versus total forms and calculated in relation to the control cells expressing empty vector. Data represent means + SD of three independent experiments and representative Western blots are shown. No statistically significant differences were detected.

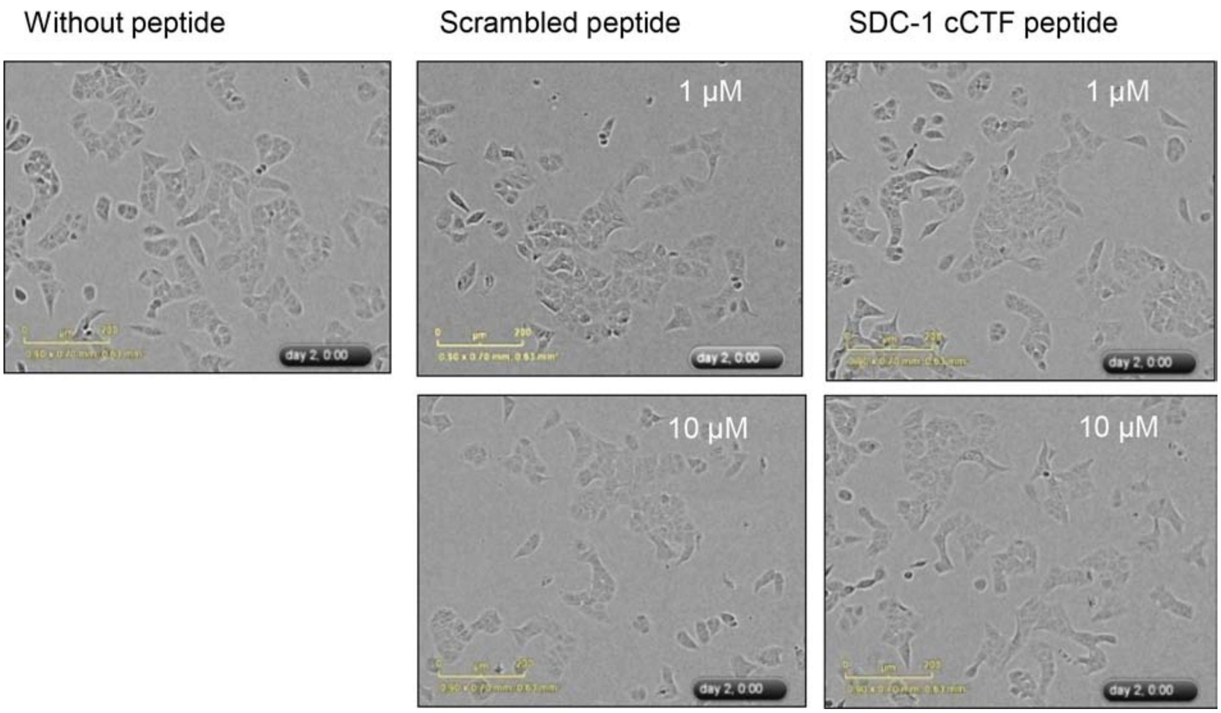

Supplementary Figure S4: Synthetic SDC-1 cCTF peptide does not affect cell morphology. A549 cells were treated with 1 or 10 μM SDC-1 cCTF peptide or the scrambled peptide with carrier reagent (Chariot delivery reagent) and analysed for morphological changes over a period of 48 h using the IncuCyte ZOOM.
